# Climatic Facilitation of the Colonization of an Estuary by *Acartia tonsa*

**DOI:** 10.1371/journal.pone.0074531

**Published:** 2013-09-30

**Authors:** Aurélie Chaalali, Grégory Beaugrand, Virginie Raybaud, Eric Goberville, Valérie David, Philippe Boët, Benoit Sautour

**Affiliations:** 1 Université Bordeaux 1, UMR 5805 EPOC, Environnements et Paléoenvironnements Océaniques et Continentaux, Station marine d'Arcachon, Arcachon, France; 2 CNRS, UMR 5805 EPOC, Environnements et Paléoenvironnements Océaniques et Continentaux, Arcachon, France; 3 Université Lille 1, UMR 8187 LOG, Laboratoire d'Océanologie et de Géosciences, Wimereux, France; 4 CNRS, UMR 8187 LOG, Laboratoire d'Océanologie et de Géosciences, Wimereux, France; 5 SAHFOS, Plymouth, United Kingdom; 6 Université Lille 1, UMR 8198 GEPV, Laboratoire de Génétique et Evolution des Populations Végétales, Villeneuve d'Ascq, France; 7 Irstea, Unité Ecosystèmes estuariens et Poissons migrateurs amphihalins, Cestas Gazinet, France; University of Hamburg, Germany

## Abstract

Global change has become a major driving force of both terrestrial and marine systems. Located at the interface between these two realms, estuarine ecosystems are probably the place where both direct and indirect effects of human activities conspire together to affect biodiversity from phytoplankton to top predators. Among European estuarine systems, the Gironde is the largest estuary of Western Europe and many studies have provided evidence that it has been affected by a variety of anthropogenic stressors such as thermal and chemical pollution, physical alterations and exploitation, especially for maritime traffic. In such a context, species introduction is also a current major issue with the establishment of strong competitive species that could lead to ecosystem reorganization with potential decrease or even disappearance of native species. In the Gironde estuary, this hypothesis was proposed for the invasive shrimp species *Palaemon macrodactylus* as a decrease in the native species abundance was observed at the same time. Although species introduction often takes place via ballast water, the influence of climate-driven changes on the establishment of new species remains a key issue. The calanoid copepod *Acartia tonsa,* observed in the Gironde estuary for the first time in 1983, have since colonized most part of the estuary, reaching a level of abundance comparable to the dominant native species *Eurytemora affinis.* In this study, using both the concept of the ecological niche *sensu* Hutchinson (fundamental and realized niches) and statistical models, we reveal that the dynamics of the colonization of *A. tonsa* was facilitated by environmental conditions that have become closer to its environmental optimum with respect to temperature and salinity.

## Introduction

Marine biodiversity and ecosystems are being altered by many human-induced factors including overexploitation of marine resources [1], [2], chemical pollution and physical alterations [3], eutrophication and invasion of exotic species [4]–[6]. Fisheries have affected the marine environment through both direct and indirect effects [1], [2], [7]. After habitat degradation, the introduction of non-native species in an environment is a major cause of extinction [8], [9]. So far, marine invasions have been less investigated although their magnitude and frequency may lead to profound changes in ecosystem functioning and biological community structure [4], [10], [11].

Estuaries, located at the interface between terrestrial and marine realms, constitute habitat for many species or even nursery, refuge and growth areas [12]–[14]. However, these ecosystems are being affected by human activities such as fishing, polluting, and maritime traffic [15], [16]. For example, maritime traffic increases the number of invasive species (e.g. copepods [17]; shrimps [18]) potentially transported via ballast waters. Survival of these species depends on environmental conditions within their new ecosystem and climate change may influence the establishment of alien species [19]. For example, Raitsos et al. [20] showed that the appearance of benthic and pelagic tropical species in the eastern part of the Mediterranean Sea was highly positively correlated with sea surface warming.

When they manage to survive, invasive species may impact their new ecosystem by affecting both its structure and functioning with implications for the structure and function of the entire food web [17], [18], [21]. For example, the establishment of the invasive copepod species *Acartia tonsa* in the oligo-mesohaline zone of the Gironde estuary led to a phenological shift in the native copepod *Acartia bifilosa* production period being advanced one month earlier in the year [17]. *Acartia tonsa* also invaded the estuary of Bilbao and altered the spatial distribution of the native copepod *Acartia clausi* [21].

In this study, we used the concept of the ecological niche *sensu* Hutchinson (fundamental and realized niches) to investigate the drivers involved in the colonization of *A. tonsa*. We considered two climate-driven parameters, temperature and salinity, for their well-known influence on biological processes. Indeed, temperature controls the kinetics of many metabolic reactions (e.g. speed of enzymatic reaction, increase in metabolism; Arrhenius [22]) and influences species reproduction, locomotion, feeding rates and interaction between species [23]–[25]. Salinity may induce potential osmotic stress, increasing the risk of species mortality [26]. Considering these two parameters, we evaluated the importance of climate change in the colonization of the Gironde estuary by the copepod *Acartia tonsa*. We applied a double modeling approach, based on both realized and fundamental niches, to provide evidence that the successful establishment of *Acartia tonsa* was the result of changes in both temperature and salinity.

## Materials and Methods

### Study area

The Gironde estuary (latitude 45°20'N, longitude 0°45′W), a 70-km long estuary formed by the junction of the Dordogne and Garonne rivers [27], is the largest south-western European estuary. With a mean suspended mater concentration higher than 500 mg.L^−1^ [28], [29], it is one of the most turbid European estuaries. The Gironde is characterized by a strong spatial and temporal variability in both physical and chemical properties [30]. In addition to the natural variability, this ecosystem is also subjected to anthropogenic pressures including climate changes [17], [30]–[33]. Among the other anthropogenic pressures, the maritime traffic (Bordeaux harbor; hull fouling, semi-dry ballast, and ballast water) represents an important cause of species introduction [17], [18]. Therefore, the Gironde estuary can be considered as a hotspot for species introduction. The calanoid copepod species *Acartia tonsa*, first introduced in 1983 by ballast waters, is an example of species that found suitable conditions to establish in the Gironde estuary.

### Environmental and zooplankton data

**Figure 1:**
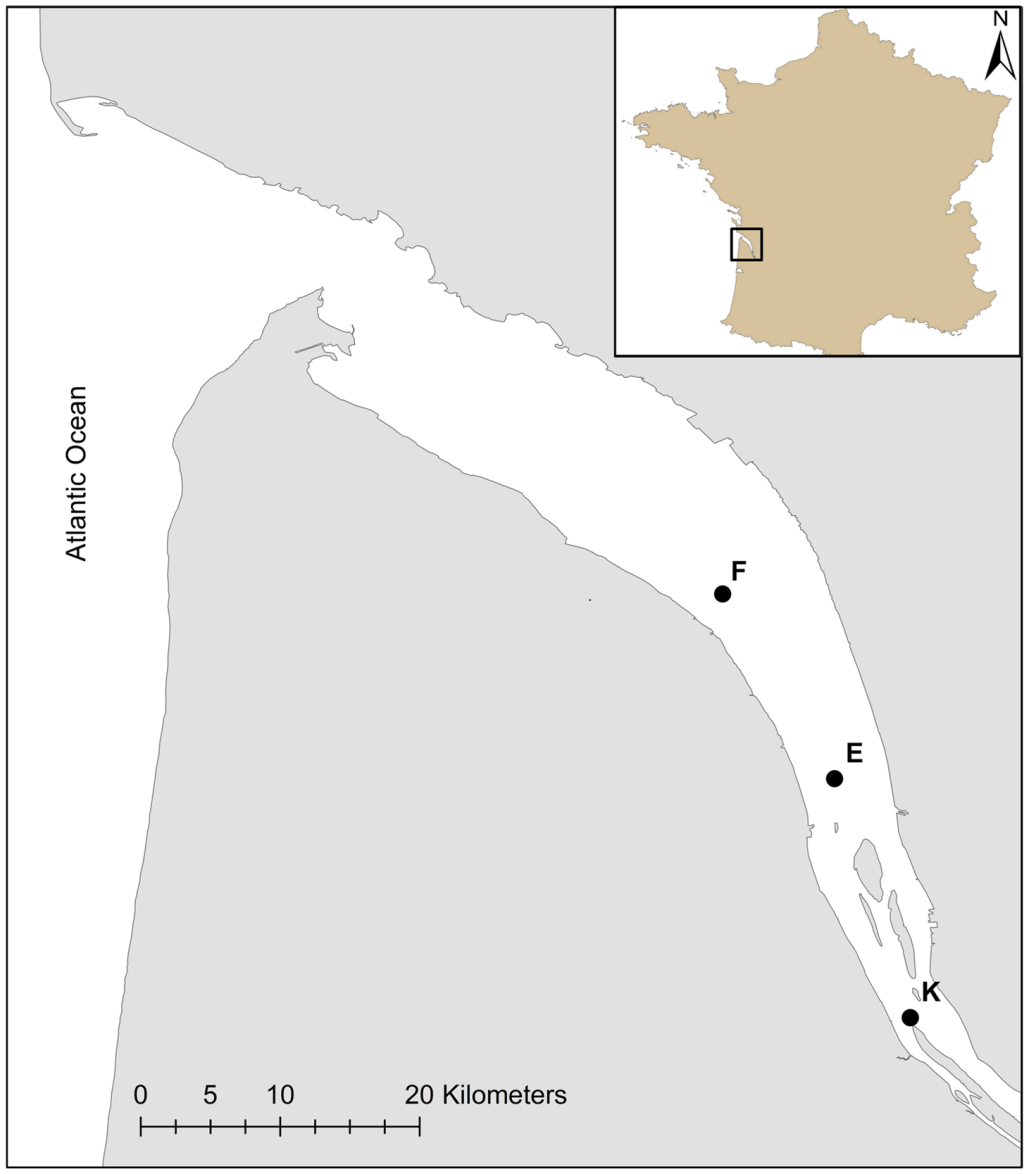
Map of the Gironde estuary with the three sampling sites (F, E, and K).

Environmental data were provided by both the “Blayais” nuclear power plant and the SOMLIT (Service d'Observation en Milieu LITtoral, a French coastal monitoring network: http://somlit.epoc.u-bordeaux1.fr; see [33] or [34]) joint monitoring. Twelve environmental parameters and the five zooplankton species abundances (including 3 copepods and 2 mysids) were monitored at monthly intervals from March 1978 to November 2009. Here, we only focused on water temperature, salinity and abundance of *Acartia tonsa* per m^3^. Samples were collected nine times a year (except for site F monitored eight times a year) at three given sites (Figure 1): (1) site E, located at 52 km from Bordeaux city, since 1978; (2) site K, located at 30 km from Bordeaux city, since 1984; (3) site F, located at 67 km from Bordeaux city, since 1992 [30]. Sampling was carried out at 1 m below the surface and 1 m above the bottom at 3 h intervals during a tidal cycle (high and low tide, flood and ebb tide).

We combined all triplets of temperature/salinity/*Acartia tonsa* abundance available in all sampling sites and obtained the data matrix: 5178 observations ×3 variables (Matrix 1). To only consider the period since species establishment, from 1999 to 2010, (the year 2011 being incomplete), we extracted data from Matrix 1. Only considering data from site E, we obtained a second matrix: 920 observations ×3 variables (Matrix 2).

### Data analyses

Based on these two matrices, we determined the ecological niche of *Acartia tonsa* as a function of both monthly water temperature and salinity. **Analysis 1** determined the realized niche, i.e. the niche including the effect of species interaction and dispersal [35], [36] by using categories of temperature and salinity in a discrete model. **Analysis 2**, applying a mixed Gaussian-linear model, also estimated species realized niche. **Analysis 3** determined the fundamental niche, i.e. the niche without the effect of dispersal or species interaction [35]. Physiological thresholds obtained from literature were subsequently implemented in the new ecological niche model NPPEN (Non-Parametric Probabilistic Ecological Niche model; [37]). This model presents several advantages: it requires presence-only data and is based on a non-parametric procedure using a simplification of the Multiple Response Permutation Procedure (MRPP) based on the Generalized Mahalanobis distance [37]. NPPEN was already applied to many marine organisms, for instance as fishes [37], [38] or benthic macrofauna [39].

The three models (discrete, Gaussian-linear and NPPEN) were then compared using Taylor diagrams (**Analysis 4;** [40]).

#### Analysis 1: Characterization of the realized niche by discrete model (DM)

**Figure 2:**
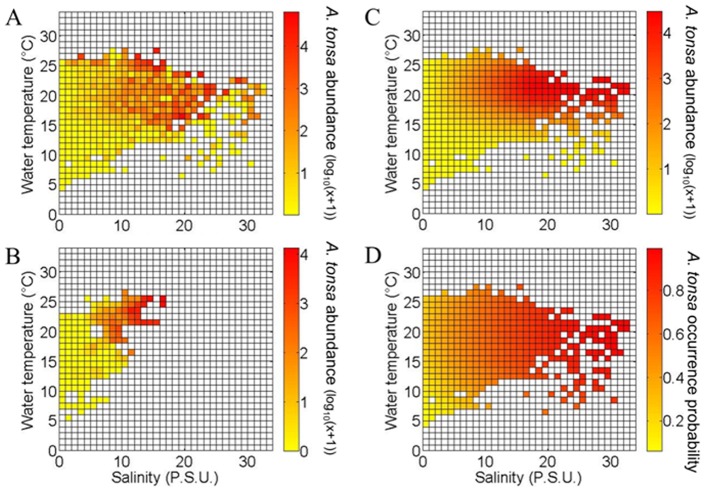
Observed and modeled ecological niche of *Acartia tonsa* in the Gironde estuary. A . Observed niche of *A. tonsa* (log_10_ (x+1)) as a function of monthly water temperature and salinity (considering all available data). **B**. Realized niche of *A. tonsa* (log_10_ (x+1)) as a function of monthly water temperature and salinity. **C**. Realized niche of *A. tonsa* (log_10_ (x+1)) as a function of monthly water temperature and salinity modeled by a mixed Gausian-linear model. **D**. Fundamental niche of *A. tonsa* (occurrence probability) modeled by the NPPEN model.

Using an approach similar to Beaugrand et al. [41], we used Matrix 2 (1999–2010 for site E) to compute the realized niche of *A. tonsa* (abundances transformed in log_10_(x+1)) as a function of monthly water temperature (from 0 to 35°C every 1°C) and salinity (from 0 to 35 psu every 1 psu). This estimation of *A. tonsa* realized niche for the period 1999–2010 was compared to the realized niche computed from all available data (for all sampling sites, i.e. Matrix 1). Using only data from site E (the longest time series) and the reference period 1999–2010, we removed any problem of circularity as the test of the model (**Analysis 4**) was performed on data from site E and other sites (F and K) for the period 1984–1998. From the knowledge of monthly water temperature and salinity of all sites, we retrieved the expected abundance of *A. tonsa* in all sites from 1984 to 2011 (see Figure 2B).

#### Analysis 2: Characterization of the realized niche by a Mixed Gaussian-linear (MGL) model

For this second analysis, we used monthly water temperature and salinity data of sampling site E for the period 1999–2010 (Matrix 2) to estimate the realized niche of *A. tonsa*. We graphically examined the relationships between the abundance of *A. tonsa* and the two environmental variables (not shown). The model was Gaussian for temperature because the examination of the data showed a maximum of abundance (as log_10_ (x+1)) flanked by two decreasing slopes. Such a function is often used to model the ecological niche [42]–[44]. As the relationship between salinity and abundance (as log_10_ (x+1)) was linear between 0 and 17 psu and leveled off after, we used a linear function between 0 and 17 psu. For salinities under 17 psu, the resulting model was the product of a linear relationship for salinity ‘S’ and a Gaussian relationship for water temperature ‘t’ (Eq. 1). For salinities over 17 psu, the model only considered the effect of temperature (Eq. 2):

For S<17 psu: 
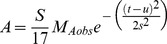
(1)


For S≥17 psu: 
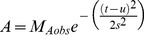
(2)with ′1/17′ the constant value of the linear relationship between *A. tonsa* and salinity S, u and s the thermal optimum and thermal amplitude of *A. tonsa*, respectively.

*M_Aobs_* is the maximum observed abundance for *A. tonsa*. The coefficients *M_Aobs_*, a, u, and s were estimated by minimizing the sum of squares of the residuals assessed by calculating the differences between observed and predicted abundance (as log_10_ (x+1)) of *A. tonsa* [42]: 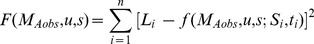
(3)with n the number of observed data (920 observations). From the knowledge of the monthly water temperature and salinity of all sampling sites, we calculated the expected abundance of *A. tonsa* for each site from 1984 to 2011.

#### Analysis 3: Characterization of the fundamental niche by the model NPPEN

To characterize the fundamental niche of *A. tonsa*, we incorporated physiological thresholds to the ecological niche model NPPEN. The model was parameterized using species physiological survival limits. According to the literature [45], [46], we determined a range of salinity between 0 and 72 psu and a range of temperature between −2 and 42°C. The eurybiontic property of the species with respect to these two parameters is exceptional. From the knowledge of the monthly water temperature and salinity of all sites, we subsequently calculated the probability of occurrence of *A. tonsa* in all sites from 1984 to 2011. For each site, the probabilities of occurrence of *A. tonsa* were then compared to the standardized abundances (between 0 and 1) of the species. Standardization was performed for each site by using the formula:
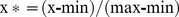
(4)with x the abundance of *A. tonsa*, min and max the minimum and maximum values of vector x, respectively.

#### Analysis 4: Models skill assessment using Taylor's diagrams

Validation and quantification of errors are important steps in any modeling study. Firstly, they provide the degree of confidence that may be given to the model for the prediction of phenomena. In addition, they can help to improve current models by revealing the mechanisms most poorly represented. There are many statistical methods to compare models and measurements and to define the level of performance of a model [47]. Each of them gives different but complementary information. Each can be used separately to test one aspect of the model, but the simultaneous use of several methods can be performed to get an overall assessment of the adjustment given by the simulation to the observed variations. This is the solution chosen by Taylor [40], which proposed to assess the performance of models by representing on a single diagram the standard deviation of the data and those of the model, the Root Mean Square Deviation (RMSD) and the correlation coefficient *r* between the model and the data. A detailed description of the Taylor diagram and formulas involved are given in ref 40 [40]. This useful tool have first been developed for atmospheric general circulation models [40] but has also been applied to physical-biogeochemical coupled models [48], [49] and generalized additive models [50]. In our case, we used Taylor's diagrams to compare the results of our three models, in each sampling site: DM' for the Discrete Model, ‘MGL’ for the Mixed Gaussian-Linear model and ‘NPPEN’ for the ecological niche model. For the comparison, all data having been previously normalized between 0 and 1 using Eq. 4.

All methods were computed using the MATLAB language.

## Results

### Estimation of both realized and fundamental niches of *A. tonsa*

The discrete and mixed Gaussian-linear models, estimating the realized niche of *A. tonsa* (Figures 2B and 2C), revealed patterns similar to the observed one (Figure 2A) despite the fact that we considered less data on Figure 2B (only data from period 1999–2010). Regarding species maximal abundances, the mixed Gaussian-linear model suggested a thermal optimum around 21.5°C (±4.5°C according to the function minimizing the sum of squares of the residuals) and salinity suitable conditions over ∼15 psu. Low species abundances were estimated for temperatures below 10°C and salinities below 3–5 psu (Figures 2A–C).

The fundamental niche of *A. tonsa* estimated by the NPPEN model was more extended than its realized niche (Figure 2D). Species environmental optima (i.e. probabilities >0.8) were obtained for a temperature ranging from 13 to 24°C and a salinity exceeding 15 psu.

### Estimation of *A. tonsa* abundance and occurrence probabilities times series

**Figure 3:**
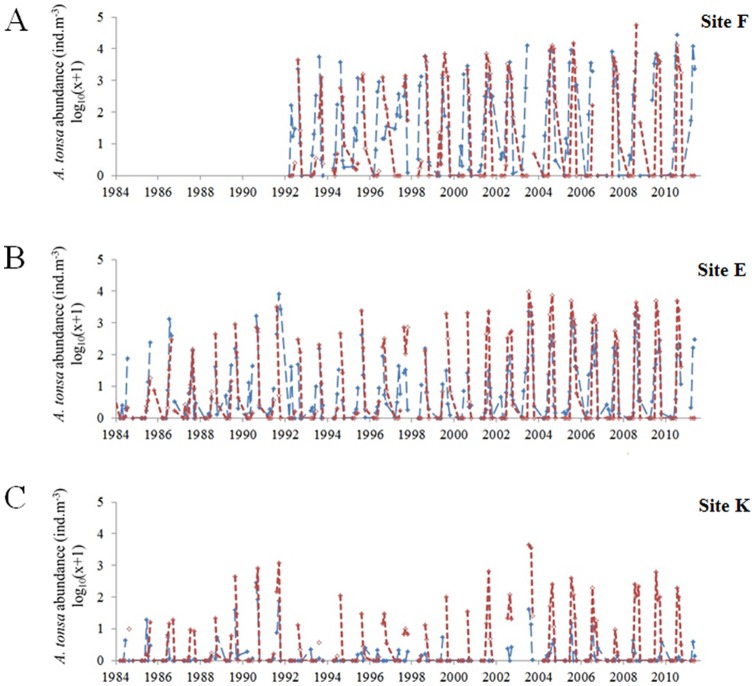
Comparison between observed (in red) and estimated (in blue) abundance of *Acartia tonsa* (log_10_(x+1)) at three sampling stations in the Gironde estuary. A . Station F. **B**. Station E. **C**. Station K. Estimated data originated from the realized niche assessed by discretization from monthly water temperature and salinity (see Fig. 2B).

**Figure 4:**
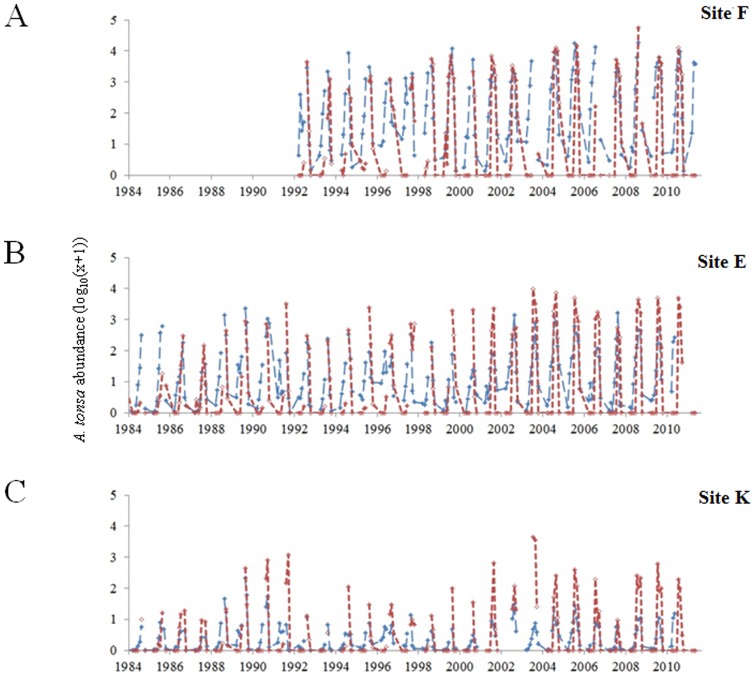
Comparison between observed (in red) and modeled (in blue) abundance of *Acartia tonsa* (log_10_(x+1)) at three sampling stations in the Gironde estuary. A . Station F. **B**. Station E. **C**. Station K. Modeled data originated from the realized niche assessed from monthly water temperature and salinity and using a mixed Gausian-linear model (see Fig. 2C).

**Figure 5:**
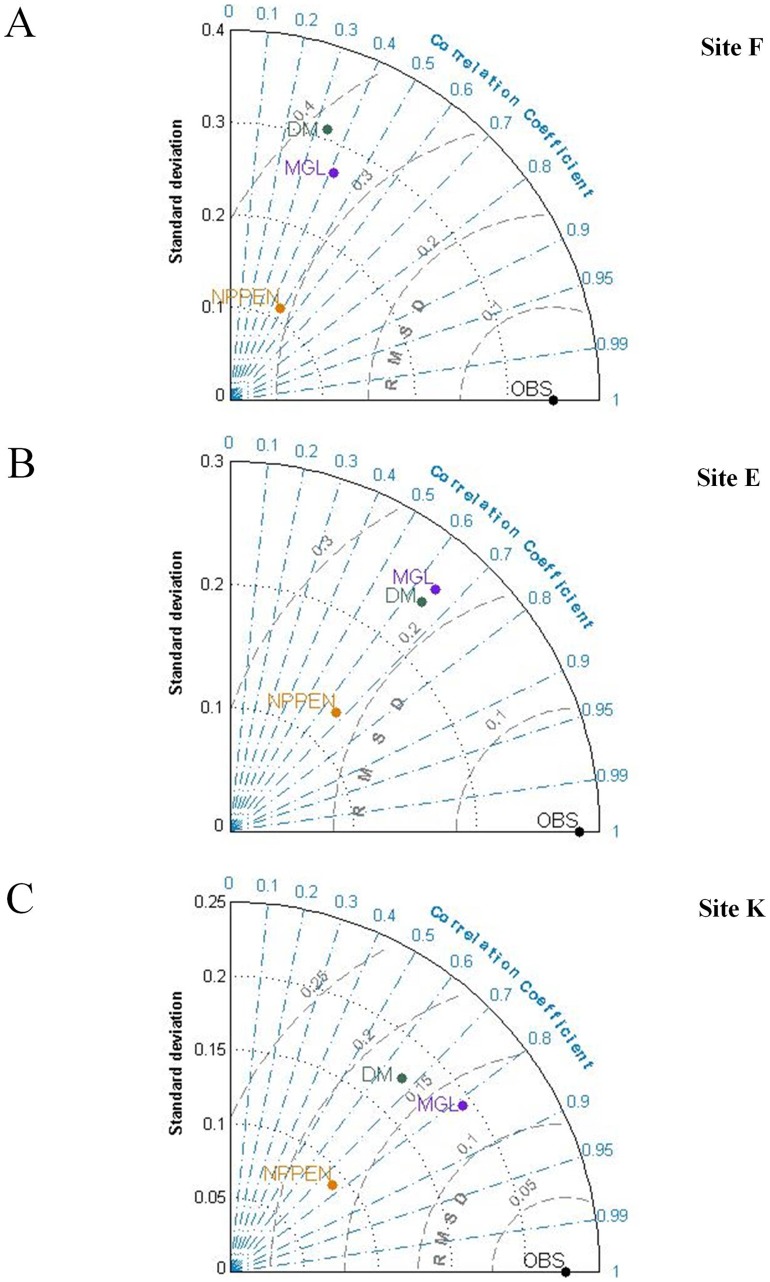
Comparison for the period 1984–1998 of the three models performances using Taylor diagrams (Taylor 2001) (‘DM’ for discrete model, ‘MGL’ for the mixed Gaussian-linear model, and ‘NPPEN’ for the ecological niche NPPEN model). **A**. Station F. **B**. Station E. **C**. Station K.

When both observed and estimated abundances of *A. tonsa* were compared for each sampling site, we found that both the discrete and mixed Gaussian-linear models reproduced well the seasonality of *A. tonsa*. However, the two models systematically underestimated species abundances at site K where the species is less abundant (Figures 3C and 4C). Regardless of the model used, both modeled and observed time series suggested a spatial saline gradient with maximal abundances observed at site F (the more downstream). Finally, both models reproduced the long-term trends observed at each site for the species (r varying between 0.35 and 0.81; Figure 5). Estimates from the two models were similar as suggested by their close positions on the Taylor diagrams (Figure 5). However, the mixed Gaussian-linear model (MGL) was more accurate than the two other models. Indeed, although the standard deviation of the discrete model (DM) was close to the observations (only for sampling site F, σ_DM_  = 0.32 and σ_OBS_  = 0.35 respectively; Figure 5A), the Pearson correlation coefficients were systematically higher for the mixed Gaussian-linear model than for the discrete model while the RMSD were low.

**Figure 6:**
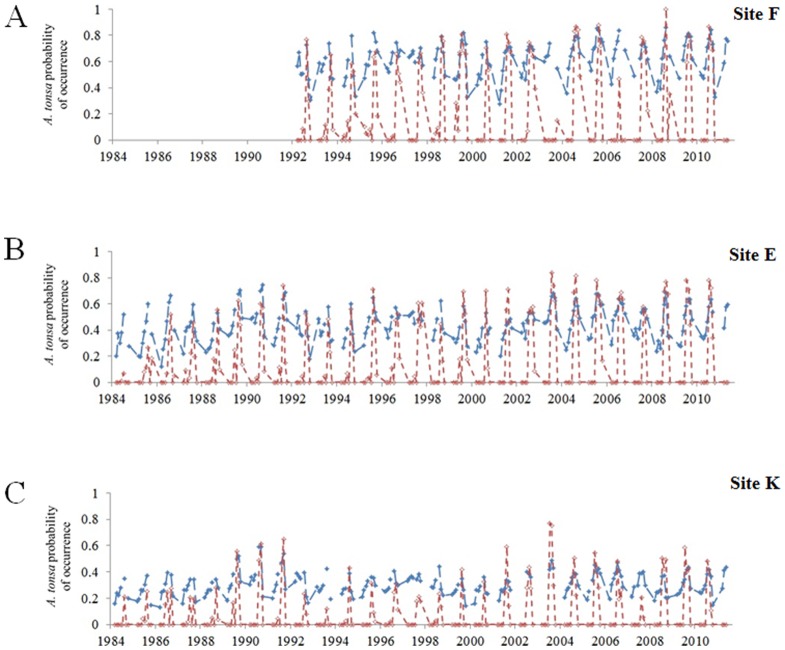
Comparison between observed (in red) and modelled (in blue) abundance of *Acartia tonsa* (log_10_(x+1)) at three sampling stations in the Gironde estuary. A . Station F. **B**. Station E. **C**. Station K. Modeled data originated from the NPPEN model (see Fig. 2D).

Year-to-year changes in the probabilities of occurrence of *A. tonsa*, assessed by the NPPEN model, showed a phase of increasing probabilities from 1983 (first observation of the species) to ∼1992 in both sites E and K. Although the seasonality was detected, the pattern was less clear than the pattern retrieved from both the discrete and mixed Gaussian-linear models. However the NPPEN model estimated a spatial distribution of *A. tonsa* in accordance with the salinity gradient: higher probabilities were estimated for the site F (Figure 6A), intermediate for site E (Figure 6B) and low probabilities for site K (Figure 6C). The RMSD and correlation coefficients between observed (i.e. abundances having been standardized) and estimated times series from the NPPEN model were similar to those of the empirical models (‘DM’ and ‘MGL’; Figure 5). Its position away from the observations on Taylor diagrams was due to the low standard deviations (σ_NPPEN_ ≤0.13) of the NPPEN model. One explanation could be that the occurrence probability range was already between 0 and 1 before normalization.

### Relation between hydro-climatic evolution and *A. tonsa* abundance

**Figure 7:**
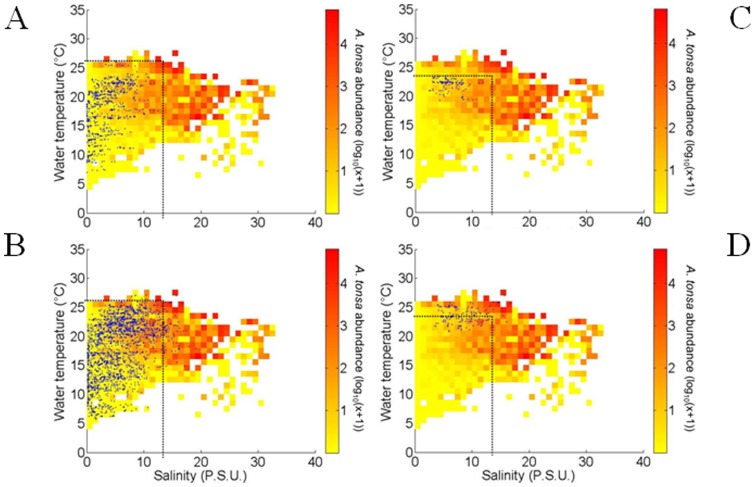
Monthly water temperature and salinity of each record of *Acartia tonsa* at Station E. A . Occurrences of *Acartia tonsa* (blue dots) for period 1978–1983 (all months). **B**. Occurrences of *Acartia tonsa* (blue dots) for period 1978–1983 (August). **C**. Occurrences of *Acartia tonsa* (blue dots) for period 1984–2011 (all months). **D**. Occurrences of *Acartia tonsa* (blue dots) for period 1984–2011 (August). The estimated realized niche was superimposed (see Fig. 2A). Both temperature and salinity maxima from period 1978–1983 were also superimposed as black dotted lines.

The monthly temperature and salinity associated with each sampling of *A. tonsa* at site E for the period 1978–1983 were characterized by maximal values of ∼26°C and ∼ 14 psu (and 23.5°C and 13.5 psu for August; Figure 7 C). We recall here that we only disposed of data before 1983 for sampling site E. Changes in both salinity and temperature in the Gironde estuary (blue dots, Figure 7) between the two periods, before (1978–1983, Figure 7A) and after the apparition of *A. tonsa* (1984–2011, Figure 7B) highlighted the influence of warming and marinisation processes; the changes in environmental conditions being associated with higher estimated abundance of *A. tonsa* (Figure 7). Comparable results with the NPPEN model (results not shown) were observed. Although the estimated probabilities of occurrence of *A. tonsa* were low for the period 1978–1983 (between 0 and 0.4), high probabilities (between 0.7 and 1) were observed from 1984 onwards.

## Discussion

Brylinski [51] first presented a map of the spatial distribution of the copepod *A. tonsa* in Europe. Originating from the North American coast, *A. tonsa* could have been transported by maritime traffic to Europe. According to David et al. [17], the copepod was observed in the Gironde estuary for the first time in 1983. Many studies have been conducted on the ecology of the species to assess its physiological traits [45], [52]–[58]. All the authors defined *A. tonsa* as a euryhaline and eurytherm species with a very large range of tolerance measured by both *in situ* and laboratory experiments [45]. These two properties explain its ubiquity in many Atlantic Ocean estuaries [45], [46], [51]. Gonzales [46] evaluated species critical thermal maximum and lethal temperature. By gradually increasing temperature until “uncoordinated” movements of the species, a critical thermal maximal value around 37°C (50% of individuals being able to tolerate a temperature of 37°C for 4 h) was evaluated and a lethal temperature near 41–46.3°C was assessed. The species was able to tolerate a wide range of temperatures between −1°C and 32°C, Gonzales [46] collected several individuals in the Narragansett Bay (Rhode Island, USA) at temperature of about −1°C. Cervetto et al. [45] experimentally characterized both the maximum and minimum values of salinity in which *A. tonsa* could survive. They estimated an impressive range between ∼1 and 72 psu with an optimal adaptation of the species between 15 and 22 psu for copepodites. These results were in accordance with previous observations [59], which found a maximal salinity threshold of 25 psu for nauplii stages. This upper value would limit the colonization of marine waters by the early stages of development of *A. tonsa,* these stages being more sensitive than the adults to changes in salinity [59].

Our estimations of both fundamental and realized niches are therefore in accordance with these experimental measurements. Estimated optimal salinity conditions were observed for values over 15–17 psu and a maximal value of 34 psu was observed in the estuary for the period 1999–2011. Moreover, our results estimated low abundances of *A. tonsa* for a salinity value under 3–5 psu. These results are supported by previous studies on species eggs quiescence and hatching success. Hojgaard et al. [53] showed that a strong quiescence process was induced at 0 salinity (and at both 17 and 25°C) and Holste and Peck ([54]; see also Peck and Holste [56]) observed an asymptotical increase in eggs hatching success for salinity between 0 and 17 psu, a maximum being observed between 17 and 25 psu.

Regarding temperature, we estimated an optimal value around 17–25°C with all modeling approaches and an abundance reduction being estimated under a temperature value of 10°C for empirical models and 13°C for the NPPEN model. However this species is also known to potentially produce diapause eggs (or low quality eggs; Zillioux and Gonzalez [60]), at a thermal threshold experimentally estimated at about 10–13°C. Under this minimal temperature value, species may produce diapause eggs and no hatching was observed for eggs incubated at low temperatures (<12°C) by Holste and Peck [54] and interestingly a maximum was observed between 22–23°C.

When the realized niche of *A. tonsa* was compared to its fundamental niche (Figures 2A, B, C *versus* 2D), we noticed some differences, especially for species occurrence at low temperatures (<13°C). These temperature values, mainly corresponding to winter conditions, are associated to species absence from samplings (see [30], [61]) even if the NPPEN model did not estimate null probabilities of species occurrence (with all salinity values associated; from 0 to ∼30 psu). However, the success of an invader population over time can not only be linked to abiotic parameters such as temperature or salinity. Biotic resistance exerted by native species can also possibly play a role and induce some differences between invader's realized and fundamental niches [62]–[64]. Indeed, the native copepod species *Eurytemora affinis* has a production period centered around March [30]. *E. affinis* would be favored at this period when the Maximum Turbidity Zone (‘MTZ’, located upstream during summer and more downstream during both autumn and winter; Sottolichio & Castaing [65]) is particularly important because this species is known (i) to be able to feed on detritus and detrital vegetal matter in contrast with *A. tonsa* [17], and (ii) to have a higher ability to select its prey among inorganic matter compared to *Acartia* spp. [66]. Thus, the NPPEN model would have not taken into account the presence of *E. affinis*. This hypothesis may be especially true for salinities between 0 and 18 psu (conditions encountered in the oligo-mesohaline estuarine regions). Regarding higher salinities, other species are present – some neritic copepod species -, that also have not been considered in the NPPEN model. Moreover, some of these species have recently been proved to significantly progress in the estuary in response to the estuary marinisation [67]. In conclusion, the physiological niche of *Acartia tonsa* estimated by the NPPEN model only reflected what would be the fundamental niche if no other parameter was acting, for example, with no predation, resource or space competition. This may also explain the difference observed between species realized and fundamental niches.

Considering species temporal variability with only 2 climate-driven parameters, we estimated values of *A. tonsa* abundances at each site. Although our models underestimated species abundance especially at the more upstream site, the seasonality of *A. tonsa* was well reproduced with a production peak around August. This result was in accordance with species seasonal pattern characterized by an autumnal peak of abundance regardless the time series period or site (see [17]) and with other studies in many North European estuaries (the Scheldt, [68]; the Ems, [69]). Although a temporal lag is sometimes noticed between times series of modeled and observed data, no lag was detected in the present study. That explains why some models include a temporal lag to account for a development (generation time) of a species. Our model also estimated a spatial distribution responding to the estuary haline gradient with maximal abundances estimated at the more downstream site. This result was not surprising as *A. tonsa* was observed in the polyhaline area of the Gironde estuary a long time before it was first recorded in the oligo-mesohaline area in 1983 [17]. This result could be more investigated by a study on the distribution of *A. tonsa* as a longitudinal shift in the distribution of its congeneric species, *A. bifilosa*, was already noticed by Chaalali et al. [67] during the same period (from 1975 to 2003).

Since the first record of *A. tonsa* in the Gironde estuary in 1983, species abundance substantially increased [17]. After 1999, this copepod became more abundant than its autochthonous congeneric species, *A. bifilosa* and even reached abundances comparable with the dominant native species *E. affinis* [17], [30]. The colonization process of the species took place in different phases: from 1978 to 1982, *A. tonsa* was absent; from 1983 to 1987, species was sporadically observed; from 1988 to 1992, the calanoid was present in low abundance; from 1993 to 1998, the species exponentially increased; and since 1999 high abundances of *A. tonsa* are regularly observed [17]. Regarding the NPPEN estimations, similar long-term trends were noticeable as we observed an increasing trend in estimated probabilities of occurrence; higher probabilities being estimated toward the end of the time series, reflecting the establishment of the species. However, the NPPEN model did not reproduce/estimate the seasonality of *A. tonsa* as well as the two empirical models. We suspect that one of the possible explanations could be linked/due to the parameterization of species physiological niche. The limit ranges of temperatures and salinities reported in the literature for species survival were different from the environmental conditions experienced by *A. tonsa* in the estuary. Indeed, the upper and lower limit values of temperature and salinity measured in the estuary during the study period ranged from 4 to 28°C for temperature (vs. −2 and 42°C reported in the literature as thermal limits; [46]) and from 0 to 33 psu for salinity (vs. 0 and 72 psu [45]).

Finally our results suggest that both warming and marinisation of the Gironde estuary between the beginning (1978–1983) and the end (1984–2011) of the studied period, facilitated the establishment of an invasive species, in accordance with Raitsos et al. [20] observations. Changes at a seasonal scale provided similar results (results not shown), comforting previous studies already pointing out strong links between temperature or salinity and *A. tonsa* abundances [17], [21]. Our study also highlights it is important to consider coupled/mixed approaches to deal with both species fundamental (i.e. physiological) and realized niches. Nevertheless previous studies (dealing with ecological niches modeling) only focused on realized niches of species (see Hirzel et al. [70] or Helaouët & Beaugrand [71]), regardeless their fundamental niches. Recent works also pointed out the importance of considering both niches [43]. As an example, Helaouët & Beaugrand [43] showed a close relationship between *Calanus finmarchicus* fundamental and realized niches that remained constant at both biogeographical and decadal scales. They observed that changes in environmental forcing propagated from the physiological to the macroecological level and, projected a potential poleward shift in species spatial distribution in the North Atlantic over the 21^st^ century. Such approaches are relevant to examine the habitat suitability of species (Diekmann et al. [72]) and to improve our knowledge about the ecology of species [43]. In the pursue of our study on a species providing a new food resource for fish and shrimps juveniles [73], there is a necessity of further investigations on species future evolution in the Gironde system in response to different climate scenarios [37]. Regarding the use of the NPPEN model for invasive species, such studies would need to integrate co-occurring species as invasions could dramatically affect other species [74] and some possible consequences were already observed [17], [67]. Such predictions have indeed to be carefully considered as non-linear evolutions may occur as some abrupt shifts were previously documented (concerning other species) and make predictions difficult [31], [67]. Moreover, while species distribution models assume equilibrium - or at least pseudo-equilibrium – between the environment and observed occurrence, a non-equilibrium phenomenon is more realistic in ecology. However, such a drawback is compensated here by both our knowledge of the physiology of the species and a large-scale approach [75] which allows us to cautiously extrapolate beyond baseline conditions to project future species' range.

Another key issue would be to study the consequences of species colonization on the structure and the function of the trophic food web, a progression of marine fish species such as the sprat (*Sprattus sprattus*) or the European anchovy (*Engraulis encrasicolus*) being already documented [76]. Indeed, previous studies revealed an increase in the abundance of small pelagics in relation to global climate change (marinisation and water warming; [76]). These species are known to predate on copepods and in particular on *Acartidae* [77]–[79]. For example, the abundance of the European anchovy in the Gironde showed a confounding trend close from the exponential trend noticed on *A. tonsa* (Delpech personal communication). While our study focused on the importance of climatic facilitation, investigations on the biotic factors that influence both the spatial and temporal colonization of *A. tonsa* would also be required.
